# Development of a Prototype, Once-Daily, Modified-Release Formulation for the Short Half-Life RIPK1 Inhibitor GSK2982772

**DOI:** 10.1007/s11095-021-03059-z

**Published:** 2021-06-16

**Authors:** Debra J. Tompson, Mark Whitaker, Rennan Pan, Geoffrey Johnson, Teresa Fuller, Litza McKenzie, Vanessa Zann, Marcy Powell, Kathy Abbott-Banner, Simon Hawkins

**Affiliations:** 1 Clinical Pharmacology Modelling and Simulation, GlaxoSmithKline, Medicines Research Centre, Gunnels Wood Road, Stevenage, Hertfordshire, SG1 2NY UK; 2 Medicine Process Delivery, GlaxoSmithKline, Dave Jack Medicines Development Centre, Park Road, Ware, Hertfordshire, SG12 0DP UK; 3 Pharmaceutical Development, GlaxoSmithKline, 1250 S. Collegeville Road, Collegeville, Pennsylvania 19426 USA; 4 Development Biostatistics, GlaxoSmithKline, 1250 S. Collegeville Road, Collegeville, Pennsylvania 19426 USA; 5 Global Clinical Sciences and Delivery, GlaxoSmithKline, Medicines Research Centre, Gunnels Wood Road, Stevenage, Hertfordshire, SG1 2NY UK; 6 Quotient Sciences Limited, Mere Way, Ruddington, Nottingham, NG11 6JS UK; 7 Safety and Medical Governance, GlaxoSmithKline, 5 Moore Drive, Research Triangle Park, North Carolina 27709-3398 USA; 8 GlaxoSmithKline, 980 Great West Road, Brentford, Middlesex, TW8-9GS UK

**Keywords:** GSK2982772, modified release, once-daily, pharmacokinetics, short half-life

## Abstract

**Purpose:**

GSK2982772 is a selective inhibitor of receptor-interacting protein kinase-1, with a 2–3 h half-life. This study evaluated if a once-daily modified-release formulation of GSK2982772 could be developed with no significant food effect.

**Methods:**

Part A evaluated the pharmacokinetics of GSK2982772 following fasted single-dose (120 mg) administration of two matrix minitab formulations (MT-8 h and MT-12 h) vs 120 mg immediate release (IR) and MT-12 h with a high-fat meal. Part B evaluated once-daily MT-12 h for 3 days at three dose levels. Part C evaluated a matrix monolithic (MM-12 h) formulation at two dose levels in different prandial states.

**Results:**

All modified-release formulations dosed in the fasted state reduced maximum plasma concentration (Cmax), delayed time to C_max_, and decreased area under the curve (AUC) vs IR. When MT-12 h or MM-12 h were co-administered with a meal (standard or high-fat) C_max_ and AUC increased. Dosing MM-12 h 1 h before a standard or high-fat meal had minimal impact on exposure vs fasted.

**Conclusions:**

MT-12 h and MM-12 h provided a QD pharmacokinetic profile in the fasted state, however when MT-12 h was dosed with a high-fat meal a QD profile was not maintained. (ClinicalTrials.gov Identifier: NCT03266172).

**Supplementary Information:**

The online version contains supplementary material available at 10.1007/s11095-021-03059-z.

## Introduction

GSK2982772 is a highly selective receptor-interacting protein kinase-1 (RIPK1) inhibitor being developed for the treatment of plaque psoriasis and other inflammatory diseases. RIPK1 plays a central role in mediating cell death and inflammation and regulates proinflammatory cytokine production downstream of numerous pathways and signalling receptors, including the tumor necrosis factor family of cytokines (1). RIPK1 exerts its signalling functions through its kinase activity as well as through its scaffolding function, which facilitates other immune processes including tumor necrosis factor-mediated classical apoptosis and nuclear factor kappa-light-chain-enhancer of activated B cell signalling (1–3). Inhibitors of RIPK1 activity such as GSK2982772 are therefore being investigated in diseases linked to tumor necrosis factor activation (4).

According to the Biopharmaceutics Classification System, GSK2982772 is considered a class 2 drug substance due to its high passive permeability (> 2 × 10^−4^ cm/s) and low solubility (0.1 mg/mL) (5). Biopharmaceutics Classification System class 2 drugs are generally well absorbed, but may be subject to solubility rate-limiting absorption. For GSK2982772, there is no evidence of dissolution rate-limiting absorption up to a dose of 240 mg administered either as standard immediate-release (IR) tablets or powder-in-capsule (6,7). The pharmacokinetic (PK) profile of GSK2982772 when administered as standard tablet or powder-in-capsule is characterized by rapid absorption, with a median time to maximum concentration (T_max_) of approximately 2 h post-dose. After attainment of maximum plasma concentration (C_max_), concentrations decline rapidly until approximately 12 h post-dose, with a half-life (t_½_) of 2–3 h followed by a slower terminal phase t_½_ of approximately 5–6 h. Because most of the systemic exposure is associated with the 2–3 h t_½_, initial clinical trials conducted with GSK2982772 have used twice-daily (BID) and thrice-daily (TID) regimens with IR 60-mg tablets (8).

For chronic inflammatory conditions, a once-daily (QD) dosing option would offer greater convenience, potentially optimize compliance and therapeutic outcome, and offer a flatter concentration-time profile with lower peak to trough concentrations compared with the IR formulation. This study was conducted to evaluate the feasibility of developing a modified-release (MR) formulation of GSK2982772 with QD dosing.

**Fig. 1:**
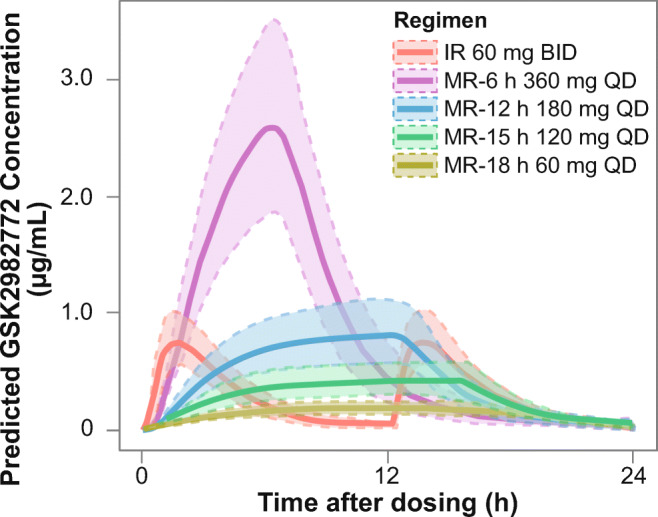
Simulations of steady-state concentration-time profiles of GSK2982772 for MR QD dosing, which provide similar trough concentrations as IR 60 mg BID, Solid line represents median predicted concentration-time profile and lighter shaded area represents the 95% prediction interval. BID, twice daily; IR, immediate release; MR, modified release; MR-6 h, modified release with 80% release at 6 h; MR-12 h, modified release with 80% release at 12 h; MR-15 h, modified release with 80% release at 15 h; MR-18 h, modified release with 80% release at 18 h; QD, once daily.

To guide the MR formulation development, simulations were conducted to predict the duration of drug release required for a QD MR formulation. The initial target was to achieve similar or lower C_max_ values and similar trough concentrations to the IR 60-mg BID dose, which was used in the initial clinical trials. The simulations used the parameters from a population PK model developed for the IR formulation. The input rate for the MR dose was assumed to be zero-order and the absorption rate constant and extent of absorption of GSK2982772 were assumed to be the same as for the IR dose. The simulations showed that the duration of drug release needed to be 12 h or more to achieve a C_max_:C_min_ ratio similar or lower than that for IR 60 mg BID (Fig. 1).

Based on the simulations, the target range of in vitro drug release duration was 80% in 10 h for “fast” release and 80% in 24 h for “slow” release. This range spanned either side of the release durations that were predicted to provide the target MR PK profile.

## Materials and Methods

### Study Design

The current study design used the Translational Pharmaceutics platform with a “formulation design space” to allow formulation adjustments in response to interim PK observations. The design space was based around a matrix MR formulation and allowed a range of dose levels and release rates (by adjustment of the percentage of polymer) to be evaluated within pre-defined extremes. Parts A and B of the study evaluated an MR matrix minitab (MT) formulation which, based on the simulations, had a target range of in vitro drug release duration of 10 h for “fast” release and 24 h for “slow” release. Each MT contained 5 mg GSK2982772 (total weight 20 mg) and a single polymer, which could be adjusted between 20% and 60% weight by weight. The dose level of GSK2982772 could be adjusted by varying the number of MTs in the capsule from 3 to 12 (corresponding to 15–60 mg GSK2982772 per capsule) or by giving multiple capsules.

Due to size restriction of the MT formulation, it was not possible to increase the polymer content beyond 60% weight by weight polymer, which resulted in release duration of 80% over 12 h (MT-12 h) for the “slow” release formulation instead of the target of 80% over 24 h. The “fast” release formulation released 80% over 8 h instead of the target of 80% over 10 h.

The maximum dose levels of the GSK2982772 MR formulation to be used throughout the study were based on the maximum IR dose that had been shown to be safe and well tolerated in healthy subjects at the time. Prior to Parts A and B, the maximum IR dose that had been administered to healthy subjects was 240 mg/day (120 mg BID). Because a concurrent high-dose PK and safety study had evaluated GSK2982772 IR dosing up to 720 mg/day (240 mg TID) (6), MR doses associated with the GSK2982772 systemic exposure for the IR 240-mg TID dose could be used in Part C. Allowances were made for the dose level to be increased if the relative bioavailability of the selected MR formulation was less than 100% relative to IR such that the daily exposure to GSK2982772 did not exceed that observed following IR administration.

**Fig. 2:**
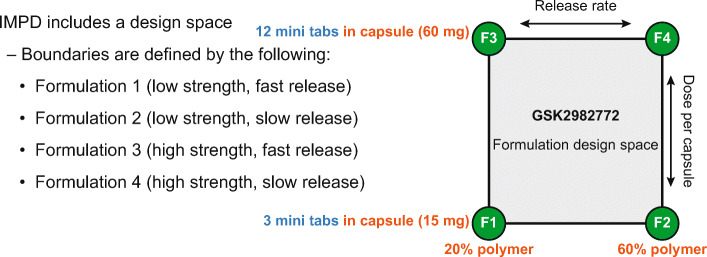
Design space for study Parts A and B.

The study was initially designed with 2 parts (A and B) that assessed the single-dose PK of GSK2982772 MR MT formulations compared with the IR formulation in healthy subjects, followed by co-administration of the selected MT formulation with a high-fat meal (Part A). It also evaluated the repeat-dose PK of MT at 3 dose levels (Part B), as well as the effect of different prandial states on single- and multiple-dose PK of MT formulations. Because Part A showed that the MT capsule formulations were susceptible to a food effect, a protocol amendment added Part C to the study to investigate a larger matrix monolithic (MM) tablet formulation. The single-dose PK profile of GSK2982772 from the MM tablet was compared with that of the IR formulation and the effect of food was also evaluated (Fig. 2).

**Fig. 3:**
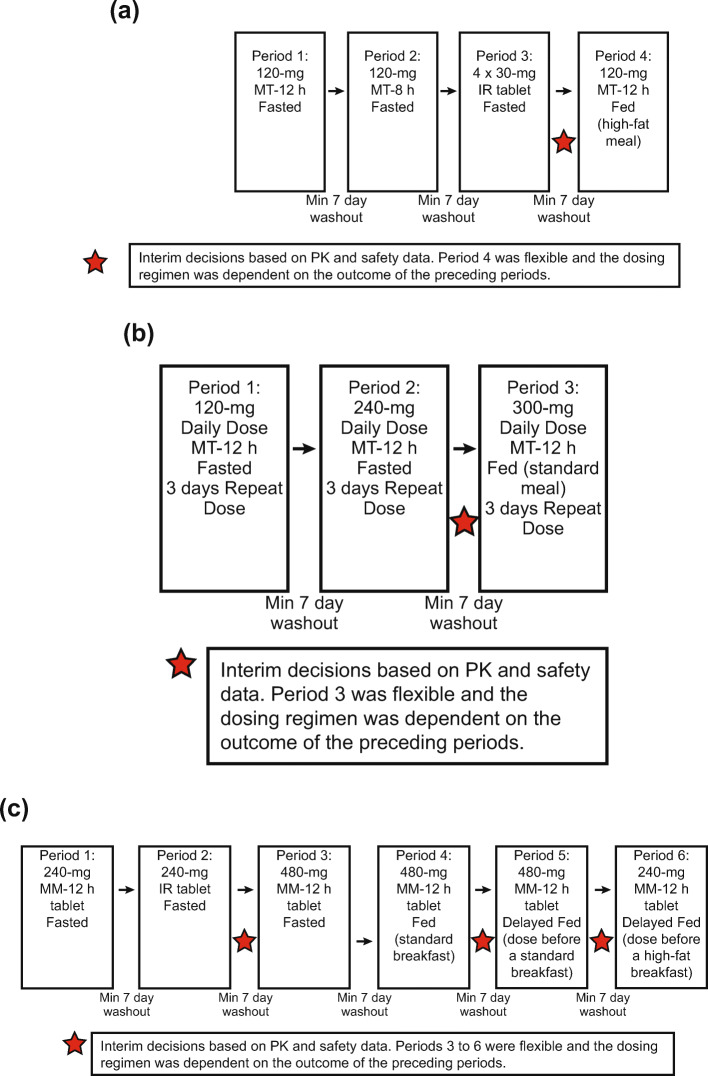
Study design. (**a**) Part A: formulation optimization and food effect. (**b**) Part B: dose ranging. (**c**) Part C: modified-release matrix monolithic tablet formulation and food effect, IR, immediate release; Min, minimum; MM-12 h, matrix monolithic with 80% release at 12 h; MT-8 h, matrix minitablet with 80% release at 8 h; MT-12 h, matrix minitablet with 80% release at 12 h; PK, pharmacokinetic.

Part A was a 6-period, sequential, 6-way, fixed-sequence study that evaluated a single oral dose of GSK2982772: Period 1: MR MT 120 mg 80% release at 12 h (MT-12 h) (fasted); Period 2: MR MT 120 mg 80% release at 8 h (MT-8 h) (fasted); and Period 3: IR tablet 120 mg (fasted). Periods 4, 5, and 6 were flexible and the dosing regimen was dependent on the outcome of Periods 1–3. In Period 4, the impact of a high-fat meal on MT-12 h was evaluated (Fig. 3a). An interim review of PK and safety following Period 4 determined the formulation (MT-12 h), dose (120 mg), dosing frequency (QD), and prandial state (fasted) for the first dosing period of Part B (see Results). Periods 5 and 6 were cancelled because no further optimization of the design space was deemed necessary.

Part B was an open-label, sequential, 3-way, fixed-sequence, repeat-dose study in which the selected MR MT formulation from Part A, MT-12 h, was evaluated after QD dosing for 3 days. Periods 1 and 2 evaluated 120 mg (fasted) and 240 mg (fasted). An interim review of PK and safety was conducted after Period 2 to decide the dose and prandial state for Period 3, which was selected as 300 mg with a standard meal (Fig. 3b).

Following completion of Parts A and B, the slowest MT formulation (MT-12 h) was shown to provide a PK profile suitable for QD dosing when administered in the fasted state or with a standard meal. However, when this formulation was administered with a high-fat meal, a positive food effect was observed, resulting in higher peak exposure and with most of the exposure in the first 12 h of dosing, which was not compatible with QD dosing. Therefore, under a protocol amendment, Part C of the study evaluated the PK and safety of a larger MM tablet (MM-12 h), which had the same in vitro 80% release duration as MT-12 h. Part C investigated whether there was solubility rate-limiting absorption at doses of up to 480 mg for the MM formulation and also assessed the impact of administration of the MM formulation in various prandial states while keeping within the exposure associated with 240 mg IR TID.

Part C was a 6-period, sequential, fixed-sequence study that evaluated single doses of MM-12 h. Periods 1 and 2 evaluated MM-12 h 240 mg (fasted) and IR tablet 240 mg (fasted), respectively. Interim reviews of PK and safety were conducted after Periods 2, 4, and 5 to decide the dose and prandial state for the subsequent period(s). Periods 3, 4, 5, and 6 evaluated MM-12 h 480 mg (fasted), MM-12 h 480 mg (standard meal), MM-12 h 480 mg (delayed standard meal), and MM-12 h 240 mg (delayed high-fat meal), respectively (Fig. 3c).

The study was approved by the South Central – Berkshire B Research Ethics Committee at the single-center participating institution and was conducted according to the recommendations of Good Clinical Practice and the Declaration of Helsinki. All subjects provided written informed consent to participate in the study.

### Study Treatment

In all 3 parts of the study (A, B, and C), subjects were admitted to the clinic the morning of the day before dosing of each inpatient period. For Parts A and C, each inpatient period consisted of 3 days and 2 nights, and for Part B, 5 days and 4 nights. Each treatment period was separated by ≥7 days of washout, and subjects were followed for at least 7 days after the last study treatment.

For fasted dosing, participants were provided a light snack the evening before dosing and then refrained from food and drink (except water) for a minimum of 10 h before dosing to approximately 4 h after dosing. The standard meal consisted of 50 g cereal with 240 mL whole milk, 1 croissant, and 1 small pot of jam. The high-fat meal consisted of 1 fried egg, 2 strips of bacon, 2 slices of buttered bread, 1 hash brown, and 240 mL whole milk. The standard meal commenced 30 min before dosing and was completed approximately 5 min before dosing. At least 90% of the meal had to be consumed for dosing to proceed. Delayed meals were taken 1 h after medication administration. For non-delayed administration, the study drug was administered 30 min after the start of a meal.

### Subjects

Male and female subjects were eligible if they were 18–65 years old, healthy (as determined by medical history, physical examination, laboratory tests, and cardiac monitoring), with body weight ≥ 50 kg and body mass index of 19.0–32.0 kg/m^2^.

### PK Sampling

Following administration of single doses of MR formulations (Part A, treatment periods 1, 2, and 4, and Part C, treatment periods 1 and 3–6), blood samples were collected pre-dose and every 2 h after dosing up to 32 h. After administration of the IR sample (Part A, treatment period 3, and Part C, treatment period 2) samples were collected pre-dose, and post-dose at 20 min, 40 min, 1, 1.5, 2, 3, and 4 h, every 2 h thereafter to 12 h, and then at 24 h. Following multiple dosing of MR MT in Part B, on days 1 and 3, PK samples were collected pre-dose and every 2 h after dosing up to 24 h. Blood samples were centrifuged within 30 min of collection, and supernatant plasma was frozen at −20°C. Plasma samples were shipped frozen on dry ice to a central laboratory, and concentrations of GSK2982772 were determined using validated analytical methods. The lower limit of quantification was 1 ng/mL.

### PK Parameters

PK parameters were calculated by standard non-compartmental analysis and using WinNonlin v8.0. The following PK parameters were determined from the plasma concentration-time data for each regimen: maximum observed plasma concentration (C_max_); time to C_max_ (T_max_); observed concentration at 24 h post-dose (C_24h_); and C_max_: C_24h_ ratio. For Parts A and C, the PK parameters also included area under the plasma concentration vs time curve (AUC) from time zero to the time of the last quantifiable concentration (AUC_[0-t]_); AUC from zero to infinity (AUC_[0-inf]_); terminal half-life (t½); relative bioavailability of test formulation vs reference IR formulation (Frel_formulation_) based on C_max_ and AUC_(0-inf)_ (or AUC_[0-t]_ when AUC_[0-inf]_ could not be derived); and relative bioavailability of fed vs fasted (Frel_FE_) based on C_max_ and AUC. In part B, additional PK parameters were determined on days 1 and 3 including dose-normalized C_max_, C_24h_, and AUC_(0–24)_; and accumulation ratio (AUC_[0–24]_ day 3 vs AUC_[0–24]_ day 1).

### Safety

Safety assessments included adverse event (AE) monitoring, clinical laboratory tests (day 1 before first treatment period, day 2 after each treatment period, at follow-up for Parts A and C and days 1 and 4, and at follow-up for part B), vital signs, electrocardiograms, and physical examination. Based on pre-clinical studies, it is unlikely that GSK2982772 is a CNS active drug; however, as a cautionary measure, the Columbia-Suicide Severity Rating Scale (C-SSRS) was used to monitor suicide risk during the multiple dosing part of the study (Part B) (9).

## Results

### Subjects

A total of 19, 10, and 16 subjects were enrolled in Parts A, B, and C, respectively, and 16 (84%), 8 (80%), and 14 (88%) subjects, respectively, completed the study. One subject in Part A withdrew because of an AE, and 6 other subjects (2 in each part) withdrew consent. In Part A, 18 of 19 subjects (95%) were white and 1 was East Asian; in Part B, 100% of subjects were white; and in Part C, 14 (88%) were white, 1 was East Asian, and 1 was South East Asian.

### Safety

In Part A, the following numbers of subjects experienced an AE: 4 participants (25%) in the 120-mg IR fasted group, 1 participant (8%) in the 120-mg MT-8 h fasted group, 5 participants (31%) in the 120-mg MT-12 h fasted group, and 2 participants (13%) in the 120-mg MT-12 h fed group. The only AEs reported by more than 1 participant in any treatment group were nasopharyngitis (2 in the 120-mg IR fasted group and 1 in the 120-mg MT-12 h fasted group), back pain (2 in the 120-mg MT-12 h fasted group and 1 in the 120-mg MT-12 h fed group), and catheter-site bruise (2 in the 120-mg MT-12 h fasted group). One subject dosed with 120-mg MT-12 h in the fasted state experienced asymptomatic bigeminy on his ECG two hours after the single dose. This resolved spontaneously but he continued to have frequent ventricular ectopic beats and was discontinued from the study at the discretion of the investigator. Twenty-eight days after this single dose he was reported to have died by asphyxia (completed suicide). This subject was discovered to have had an undisclosed suicide attempt before study enrollment. Following a single dose of the short half-life study drug GSK2982772, there was no temporal relationship to support a causal association and the death was judged by the investigator to be unrelated to study drug. Only 1 drug-related AE was reported: mild dizziness after dosing with the 120-mg IR tablet in the fasted state.

In Part B, the following numbers of subjects experienced an AE: 1 participant (10%) in the 120-mg MT-12 h fasted group, 3 (30%) in the 240-mg MT-12 h fasted group, and 1 (17%) in the 300-mg MT-12 h fed (standard) group. No AE was reported by more than 1 participant. Drug-related AEs of mild jaw pain and headache were reported by 1 participant in the 120-mg MT-12 h fasted group. The AEs resolved within 3 h of onset. There were no deaths or serious AEs in this part of the study.

In Part C, the following numbers of subjects experienced an AE: 3 participants (20%) in the 240-mg IR fasted group, 3 (20%) in the 240-mg MM-12 h fasted group, 3 (19%) in the 480-mg MM-12 h fasted group, 4 (25%) in the 480-mg MM-12 h fed (standard) group, 2 (13%) in the 480-mg MM-12 h delayed fed (standard) group, and 1 (7%) in the 240-mg MM-12 h delayed fed (high-fat) group. No drug-related AEs were reported.

No clinically important changes were reported for laboratory assessments, vital signs, electrocardiogram findings (other than for the participant with clinically significant heart rhythm changes), or suicidality in Parts A, B, or C. No reports of suicidality were identified by the C-SSRS in Part B. The C-SSRS was not used for monitoring during the single-dose parts of the study, but other than the subject (in Part A) with completed suicide who did not disclose the prior suicide attempt or a change in mood before the suicide, there were no reports of suicidality or mood change in Parts A or C.

### PK Analyses

#### Part a

**Fig. 4:**
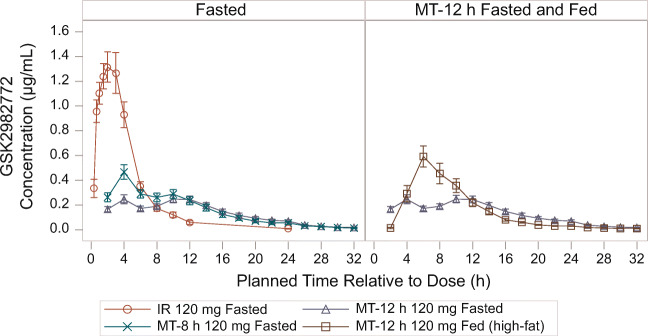
Mean (± SE) plasma GSK2982772 concentration-time profile following a single dose, by formulation and prandial state (Part A), IR, immediate release; MT-8 h, matrix minitablet with 80% release at 8 h; MT-12 h, matrix minitablet with 80% release at 12 h; SE, standard error.

**Fig. 5:**
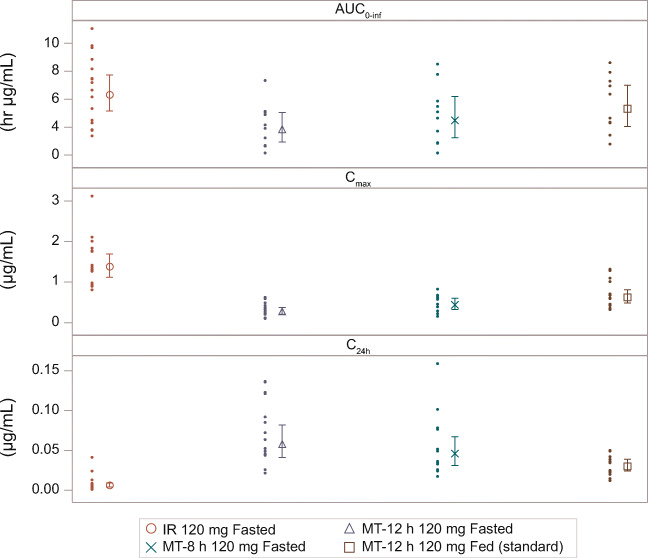
Individual subject (geometric mean [± 95% CI]) plasma GSK2982772 AUC_(0-inf)_, C_max_, and C_24h_, by formulation and prandial state (Part A). AUC_(0-inf)_, area under the plasma concentration vs time curve zero to infinity; C_24h_, concentration at 24 h post-dose; CI, confidence interval; C_max_, maximum plasma concentration; IR, immediate release; MT-8 h, matrix minitablet with 80% release at 8 h; MT-12 h, matrix minitablet with 80% release at 12 h.

When compared with the 120-mg IR formulation, administration of the 120-mg MT-8 h and MT-12 h formulations in the fasted state resulted in flatter GSK2982772 concentration-time profiles with delayed T_max_ (median T_max_ of 4 h and 10 h post-dose, respectively, vs 2 h post-dose with the IR formulation) and reduced GSK2982772 C_max_ (geometric mean ratios of C_max_ values were 32.1% and 20.4%, respectively, of IR C_max_) (Table I; Figs. 4 and 5). Geometric mean ratios of C_24h_ values were 8.6-fold and 11-fold higher, respectively, than C_24h_ for the IR formulation. The mean C_max_:C_24h_ ratios for both MT-8 h (11.2) and MT-12 h (4.3) were substantially lower than that for the IR formulation (280). Based on AUC_(0-inf)_, the bioavailability of GSK2982772 relative to the IR formulation was lower for MT-12 h (60.5%) than for MT-8 h (72.8%) (Table II).

**Table I:** Summary statistics of derived plasma GSK2982772 PK parameters after single-dose administration of 120-mg MT or IR formulation and effect of prandial state (Part A)

	AUC_(0-inf)_ (h∙μg/mL) Geometric mean (95% CI)	C_max_ (μg/mL) Geometric mean (95% CI)	C_24h_ (μg/mL) Geometric mean (95% CI)	T_½_ (h) Median (min, max)	T_max_ (h) Median (min, max)
IR fasted	6.31 (5.15–7.72)	1.38 (1.12–1.69)	0.006 (0.004–0.009)	3.34 (2.69, 6.53)	2.00 (0.67, 3.00)
MT-12 h fasted	3.85 (2.94–5.06)	0.28 (0.21–0.37)	0.06 (0.04–0.08)	3.11 (2.17, 7.96)	10.00 (2.08, 18.00)
MT-8 h fasted	4.48 (3.25–6.19)	0.44 (0.32–0.60)	0.05 (0.03–0.07)	3.51 (2.61, 7.75)	4.00 (4.0, 10.00)
MT-12 h high-fat meal	5.31 (4.04–6.99)	0.62 (0.48–0.81)	0.03 (0.02–0.04)	3.32 (2.06, 6.33)	6.00 (4.0, 10.00)

AUC_(0-inf)_, area under the plasma concentration vs time curve from 0 to infinity; C_24h_, observed concentration at 24 h post-dose; CI, confidence interval; C_max_, maximum plasma concentration; IR, immediate release; MT, matrix minitablet; MT-8 h, MT with 80% release at 8 h; MT-12, MT with 80% release at 12 h; PK, pharmacokinetic; T½, terminal half-life; T_max_, time to C_max_

**Table II:** Summary of statistical analyses of plasma GSK2982772 PK parameters assessing relative bioavailability of 120-mg MT formulation (Part A)

	AUC_(0-inf)_ Geometric Mean Ratio (90% CI)	AUC_(0–24)_ Geometric Mean Ratio (90% CI)	C_max_ Geometric Mean Ratio (90% CI)	C_24h_ Geometric Mean Ratio (90% CI)
MT-8 h fasted vs IR fasted (*N* = 13)	0.73 (0.68–0.78)	0.68 (0.64–0.73)	0.32 (0.27–0.38)	8.60 (6.31–11.72)
MT-12 h fasted vs IR fasted (*N* = 16)	0.61 (0.56–0.65)	0.58 (0.54–0.61)	0.20 (0.17–0.24)	10.97 (8.10–14.85)
MT-12 h (high-fat) vs MT-12 fasted (*N* = 16)	1.24 (1.16–1.32)	NC	2.25 (2.02–2.51)	NC

AUC_(0-inf)_, area under the plasma concentration vs time curve from 0 to infinity; AUC_(0–24)_, AUC from 0 to 24 h; C_24h_, observed concentration at 24 h post-dose; CI, confidence interval; C_max_, maximum plasma concentration; IR, immediate release; MT, matrix minitablet; MT-8 h, MT with 80% release at 8 h; MT-12, MT with 80% release at 12 h; NC, not calculated; PK, pharmacokinetic

MT-12 h was selected for the food effect arm, as it showed a flatter PK profile in the fasted state compared with MT-8 h and was therefore more suitable for the target QD dosing regimen. When MT-12 h was administered with a high-fat meal, GSK2982772 C_max_ was on average 2.25-fold higher and AUC_(0-inf)_ 1.24-fold higher, compared with administration in the fasted state, and the C_max_:C_24h_ ratio was approximately 5-fold higher in the fed state (22.4) compared with the fasted state (4.3). The PK profile of the MR MT formulation when administered with a high-fat meal was not considered suitable for QD dosing, as most of the exposure to GSK2982772 occurred within the first 12 h of dosing.

#### Part B

Since co-administration of MT-12 h with a high-fat meal in Part A did not represent the target QD profile, it was decided that the first 2 arms in Part B would evaluate 3 days of repeated dosing with GSK2982772 MT-12 h 120 mg and 240 mg QD in the fasted state. Initially, the third arm of Part B was to evaluate 360 mg QD in the fasted state which, due to the 60.5% relative bioavailability of MT-12 h vs IR, would keep daily exposure to GSK2982772 within the observed daily exposure for 240 mg IR. However, MT-12 h 300-mg QD dosing with a standard meal over 3 days was evaluated to understand what impact this food regimen would have on the PK of GSK2982772. The selection of a 300-mg dose allowed for the worst-case scenario of a 1.24-fold increase in exposure as observed with the high-fat meal.

**Fig. 6:**
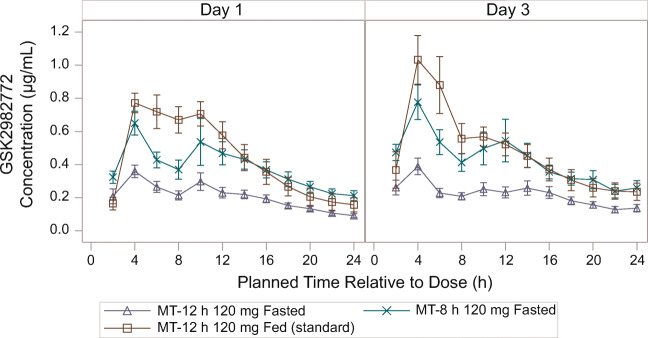
Mean (± SE) plasma GSK2982772 concentration-time profile following repeated dosing of MR MT-12 h for 3 days (Part B). MR, modified release; MT-8 h, matrix minitablet with 80% release at 8 h; MT-12 h, matrix minitablet with 80% release at 12 h; SE, standard error.

Following single and repeated oral administration of MR MT-12 h for 3 days as 120 mg fasted, 240 mg fasted, or 300 mg fed (standard meal), systemic exposure (C_max_ and AUC_[0–24]_) was similar on day 1 and day 3 for each dose group (Fig. 6; **Online Resource I and II**).

**Fig. 7:**
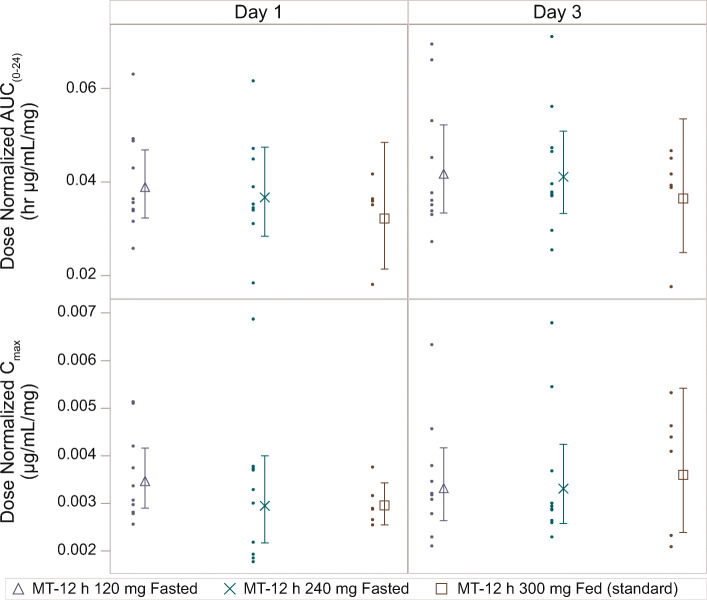
Geometric mean (± 95% CI) and individual observed dose-normalized exposure to GSK2982772 on day 1 and after repeated dosing on day 3 (Part B). AUC_(0–24)_, area under the plasma concentration vs time curve from zero to 24 h; CI, confidence interval; C_max_, maximum plasma concentration; IR, immediate release; MT-12 h, matrix minitablet with 80% release at 12 h.

Both C_max_ and AUC_(0–24)_ increased approximately linearly with dose over the dose range of 120–300 mg QD based on the similarity of the range of dose-normalized PK parameters across the doses (Fig. 7).

#### Part C

Because the MT-12 h formulation provided a suitable PK profile for QD dosing but was susceptible to a food effect with a high-fat meal, a switch from the MT formulation to an MM tablet formulation was made for Part C. Evaluation of the performance of the larger MR tablet was possible with the MM tablet as it has the advantage that a greater proportion of polymer can be added in order to further slow the release rate of GSK2982772 in future studies. To keep within the design space of the current study, the MM tablet with 80% release at 12 h (MM-12 h) was studied.

**Fig. 8:**
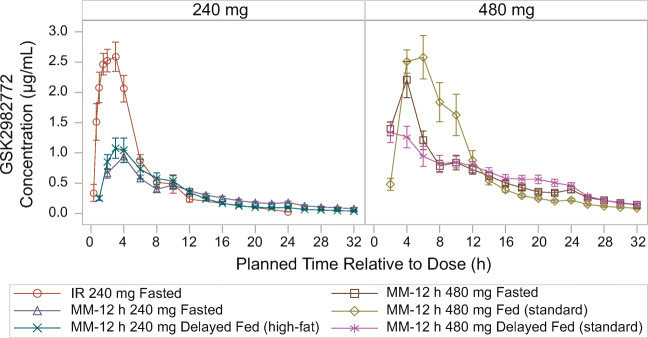
Mean (± SE) plasma GSK2982772 concentration-time profile following a single dose of MR MM-12 h (Part C), by formulation and prandial state. IR, immediate release; MM-12 h, matrix monolithic with 80% release at 12 h; MR, modified release; SE, standard error.

Following single-dose administration of 240-mg MM-12 h formulation in the fasted state, median T_max_ was delayed (4–5 h post-dose) compared with the 240-mg IR formulation (2 h) (Fig. 8 and Table III).

**Table III:** Summary statistics of derived plasma GSK2982772 PK parameters after single-dose administration of MM and IR formulations and effect of prandial state (Part C)

	AUC_(0-t)_ (h∙μg/mL) Geometric Mean (95% CI)	C_max_ (μg/mL) Geometric Mean (95% CI)	C_24h_ (μg/mL) Geometric Mean (95% CI)	T_max_ (h) Median (min, max)
240 mg IR fasted	14.62 (12.71–16.82)	2.94 (2.56–3.37)	0.03 (0.02–0.03)	2.00 (0.67, 10.1)
240 mg MM-12 h fasted	9.68 (8.24–11.36)	0.92 (0.76–1.10)	0.16 (0.12–0.21)	4.00 (2.00, 10.0)
480 mg MM-12 h fasted	20.01 (17.13–23.37)	2.01 (1.56–2.59)	0.35 (0.27–0.46)	4.00 (2.00, 12.0)
480 mg MM-12 h standard meal	22.71 (18.84–27.38)	3.15 (2.66–3.73)	0.19 (0.15–0.26)	5.02 (4.00, 10.0)
480 mg MM-12 h delayed fed standard meal	19.15 (16.44–22.30)	1.55 (1.25–1.92)	0.42 (0.33–0.54)	4.00 (2.00, 20.0)
240 mg MM-12 h delayed fed high-fat meal	9.20 (7.13–11.87)	1.06 (0.76–1.48)	0.08 (0.05–0.12)	4.00 (2.00, 10.02)

AUC_0-t_, area under the plasma concentration vs time curve for the dosing interval; C_24h_, observed concentration at 24 h post-dose; C_max_, maximum plasma concentration; IR, immediate release; MM, matrix monolithic; MM-8 h, MM with 80% release at 8 h; MM-12, MM with 80% release at 12 h; PK, pharmacokinetic; T½, terminal half-life; T_max_, time to C_max_

Geometric mean C_max_ for MM-12 h fasted was 31% of the C_max_ observed for the IR formulation and C_24h_ was 6.5-fold higher than for the IR formulation. C_max_ and AUC_0-t_ values increased approximately linearly with dose between 240 mg and 480 mg MM-12 h. The bioavailability of GSK2982772 relative to the IR formulation was 66% (**Online Resource III**).

When 480 mg MM-12 h was administered after a standard meal, a 1.57-fold increase in C_max_ and 1.14-fold increase in AUC_(0-t)_ was observed compared with fasted dosing. Delaying the meal (standard or high-fat) by 1 h mitigated the effect of food and had minimal impact on overall exposure compared with fasted dosing (**Online Resource III**).

## Discussion

GSK2982772, a RIPK1 inhibitor under development for the treatment of plaque psoriasis, has been dosed BID or TID in clinical trials as an IR formulation. This study evaluated the PK and safety of the matrix MR formulations of GSK2982772 MT and MM to determine the feasibility of developing a QD regimen to optimize patient compliance.

No new safety issues and no clinically significant findings were reported for laboratory assessments, vital signs, electrocardiograms, suicidality, or physical examinations after administration of up to 480 mg of a single dose or 300 mg QD for 3 days of an MR formulation. The overall incidence of AEs was low and all AEs resolved by the end of the study. Increased dose or duration of dosing was not associated with any clear trend in increase in number or severity of AEs.

All MR formulations tested provided flatter profiles compared with the IR formulation, as shown by delayed T_max_, reduced C_max_, and increased C_24h_ values. In Part A, there was a rank order relationship between the duration of drug release of the 120-mg MT formulations and the PK of GSK2982772. The longer release duration of MT-12 h resulted in longer T_max_, lower C_max_, higher C_24h_, and reduced AUC_(0-inf)_ compared to MT-8 h; this relationship for T_max_, C_max_, and C_24h_ was as expected, since the longer the drug release duration from the MT, the flatter the concentration-time profile. However, the overall extent of absorption was lower with the MT formulations compared with the IR (73% for MT-8 h and 61% for MT-12 h). This finding was unexpected because the in vitro duration of drug release for MT-8 h and MT-12 h is shorter than typical gastrointestinal transit time (24 h), and GSK2982772 is passively permeable, hence absorption of GSK2982772 would be anticipated along the length of the intestinal tract. However, as GSK2982772 is a Biopharmaceutics Classification System class 2 drug, its solubility may limit dissolution in the lower fluid volume associated with the colon, and thus reduce absorption there (10,11).

In Part A, Period 4, both rate and extent of absorption of GSK2982772 were increased when MT-12 h was administered with a high-fat meal compared with the fasted state. C_max_ was more than doubled (2.25-fold increase), C_max_:C_min_ ratio was increased by approximately 5-fold, and overall extent of absorption was approximately 25% higher. The observed food effect is thought to be due to increased retention time in the stomach with a high-fat meal (4–6 h) compared with the fasted state (~0.5 h). The mechanical stress in the fasted stomach is relatively low and a matrix MR tablet is likely to pass through intact. However, when a matrix MR tablet is administered with a high-fat meal it is susceptible to digestive mechanical stress due to the longer retention time in the stomach (12,13). When the stomach empties, the dissolved drug becomes available for absorption. Although exposure increased in the fed state, dose dumping was not observed, indicating that the formulation maintained some MR functionality.

For all 3 dose levels in Part B, there was minimal accumulation between day 1 and day 3. The PK appeared linear over the dose range of 120–300 mg, with similar dose-normalized C_max_, AUC, and C_24h_ across all doses and prandial states, which suggests a standard meal had no impact on the PK when administered with MT-12 h.

In Part C, the formulation was switched from matrix MTs in capsules to a larger matrix monolithic tablet, which was considered more suitable for any future clinical studies that may be considered with a matrix formulation. The duration for 80% release of GSK2982772 for MM-12 h was the same as MT-12 h. Because both formulations use the same matrix technology, the food effect observed with the high-fat meal in Part A with the MT-12 h formulation was also anticipated with the MM-12 h formulation. The primary aims of Part C were to determine whether there was dissolution rate-limiting absorption at MM-12 h doses of up to 480 mg and whether food effects were reduced by administration of MM-12 h with a standard meal or a delayed standard or high-fat meal.

The bioavailability of 240 mg MM-12 h relative to 240 mg IR (66%) was similar to the relative bioavailability of 120 mg MT-12 h (61%), although the reduction in C_max_ for MM-12 h (31% of IR) was not as great as for MT-12 h (20% of IR). The C_max_, AUC, and C_24h_ appeared to be approximately linear between 240 mg MM-12 h and 480 mg MM-12 h, indicating no solubility rate-limiting absorption at the 480-mg dose. Comparison of 240 mg and 480 mg MM with 120 mg MT also indicated linear PK.

The impact of food on the MM-12 h formulation was assessed to help guide dosing recommendations for future clinical trials. Dosing 480 mg with a standard breakfast resulted in a 57% increase in C_max_ and minimal increase in AUC_(0-t)_ (14%) compared with fasted dosing. However, dosing this formulation 1 h before a standard or high-fat meal had minimal impact on overall exposure compared with fasted dosing.

In summary, this study demonstrated that a QD profile could be achieved with GSK2982772 despite its short effective half-life. The results of this study were used to support the design of the subsequent study which was conducted utilizing a GSK proprietary modified release technology (DiffCORE™) which has previously shown robustness against food effects. This subsequent study also evaluated the impact of longer duration of drug release (up to 80% release at 18 h) and an assessment of PK linearity up to a dose of 960 mg.

## Conclusions

Single and repeat doses of GSK2982772 MR (MT and MM formulations) were generally well tolerated in healthy subjects. In vitro release of 80% GSK2982772 over 12 h with either MT or MM formulation provided a QD dosing PK profile in the fasted state. The MR formulations performed as expected with delayed T_max_, reduced C_max_, and higher C_24h_ values. The MT-12 h formulation when administered with a high-fat meal increased both C_max_ and AUC and resulted in a PK profile that was not consistent with QD dosing. Administration of GSK2982772 as 240-mg MM tablets with a standard meal or 1 h before a meal (standard or high-fat) resulted in a PK profile suitable for QD dosing. The PK of MR formulations were approximately linear up to doses of 480 mg, indicating no solubility rate-limiting absorption.

### Acknowledgments and Disclosures

All listed authors meet the criteria for authorship set forth by the International Committee for Medical Journal Editors. The authors wish to thank Monica Simeoni, who conducted the simulations that guided us on the target release rate. They also thank Quotient Sciences, where the study was conducted, and The Doctor’s Laboratory and Covance Laboratories for lab support. Editorial support (assembling tables and figures, collating author comments, copyediting, fact checking, and referencing) and graphic services were provided by AOIC, LLC, and were funded by GSK. **Debra J. Tompson**: contributed to the conception or design of the study; contributed to the acquisition of the data; contributed to the data analysis or interpretation; provided critical review and final approval of the publication. **Mark Whitaker**: contributed to the conception or design of the study; contributed to the data analysis or interpretation; provided critical review and final approval of the publication. **Rennan Pan**: contributed to the conception or design of the study; provided critical review and final approval of the publication. **Geoffrey Johnson**: contributed to the conception or design of the study; contributed to the data analysis or interpretation; provided critical review and final approval of the publication.

**Teresa Fuller**: contributed to the conception or design of the study; contributed to the acquisition of the data; contributed to the data analysis or interpretation; provided critical review and final approval of the publication. **Litza McKenzie**: study principal investigator; responsible for clinical conduct of the study; contributed to data analysis or interpretation; provided critical review and final approval of the publication.

**Vanessa Zann:** contributed to the data analysis or interpretation; provided critical review and final approval of the publication. **Marcy Powell**: contributed to the conception or design of the study; contributed to the data analysis or interpretation; provided critical review and final approval of the publication. **Kathy Abbott-Banner**: provided critical review and final approval of the publication. **Simon Hawkins**: provided critical review and final approval of the publication. This study (NCT03266172 available from **www.clinicaltrials.gov**) was funded by GlaxoSmithKline (GSK). DJT, MW, RP, GJ, TF, MP, KA-B, and SH are employees of and hold equity stock in GlaxoSmithKline. LM and VZ are employees of Quotient Sciences Limited, which received funding to conduct the study. The study was approved by the South Central – Berkshire B Research Ethics Committee at the single-center participating institution and was conducted according to the recommendations of Good Clinical Practice and the Declaration of Helsinki. Informed consent was obtained from all individual subjects included in the study. Anonymized individual participant data and study documents can be requested for further research from www.clinicalstudydatarequest.com.

## Supplementary Information

ESM 1 (DOCX 50 kb)

## Abbreviations

0–24: Plasma concentration vs time curve from zero to 24 h
0-inf: Plasma concentration vs time curve from zero to infinity
AE: Adverse event
AUC: Area under the curve
BID: Twice daily
C_24h_: Concentration at 24 h post-dose
C_max_: Maximum plasma concentration
IR: Immediate release
MM: Matrix monolithic
MR: Modified release
MT: Minitablet
PK: Pharmacokinetic
QD: Once daily
RIPK1: Receptor-interacting protein kinase-1
T_max_: Time to maximum concentration
