# Supplementary material for: Development of a Prototype, Once-Daily, Modified-Release Formulation for the Short Half-Life RIPK1 Inhibitor GSK2982772
Source: Pharm Res. 2021 Jun 16;38(7):1235–45. doi: 10.1007/s11095-021-03059-z (PMC8292240; doi:10.1007/s11095-021-03059-z)
Supplement: Supplementary file 1 — (DOCX 50 kb) [file 11095_2021_3059_MOESM1_ESM.docx]

***Pharmaceutical Research***

# Electronic Supplementary Material

**Development of a Prototype, Once-Daily, Modified-Release Formulation for the Short Half-Life Molecular Entity GSK2982772**

Debra J. Tompson^*1^, Mark Whitaker^2^, Rennan Pan^3^, Geoffrey Johnson^4^, Teresa Fuller^5^, Litza McKenzie^6^, Vanessa Zann^6^, Marcy Powell^7^, Kathy Abbott-Banner^8^, Simon Hawkins^8^

^1^Clinical Pharmacology Modelling and Simulation, GlaxoSmithKline, Medicines Research Centre, Gunnels Wood Road, Stevenage, Hertfordshire SG1 2NY, UK

^2^Medicine Process Delivery, GlaxoSmithKline, Dave Jack Medicines Development Centre, Park Road, Ware, Hertfordshire SG12 0DP, UK

^3^Pharmaceutical Development, GlaxoSmithKline, 1250 S. Collegeville Road, Collegeville, PA 19426, USA

^4^Development Biostatistics, GlaxoSmithKline, 1250 S. Collegeville Road, Collegeville, PA 19426, USA

^5^Global Clinical Sciences and Delivery, GlaxoSmithKline, Medicines Research Centre, Gunnels Wood Road, Stevenage, Hertfordshire SG1 2NY, UK

^6^Quotient Sciences Limited, Mere Way, Ruddington, Nottingham NG11 6JS, UK

^7^Safety and Medical Governance, GlaxoSmithKline, 5 Moore Drive, Research Triangle Park, NC 27709-3398, USA

^8^GlaxoSmithKline, 980 Great West Road, Brentford, Middlesex TW8-9GS, UK

**Corresponding Author:**

*Debra J. Tompson, MSc

GlaxoSmithKline

Email: debra.j.tompson@gsk.com

**Online Resource I.** Summary of statistical analyses of derived plasma GSK2982772 PK parameters after repeat-dose administration of 120 to 300 mg MT-12 h (Part B)

|  | AUC_(0-24)_ (h∙μg/mL)  Geometric Mean (95% CI) | C_max_ (μg/mL)  Geometric Mean (95% CI) | C_24h_ (μg/mL)  Geometric Mean (95% CI) | T_max_ (h)  Median (min, max) |
| --- | --- | --- | --- | --- |
| 120 mg fasted  Day 1  Day 3 | 4.67 (3.88–5.63)  5.01 (4.01–6.26) | 0.42 (0.35–0.50)  0.40 (0.32–0.50) | 0.08 (0.06–0.12)  0.12 (0.08–0.18) | 4.06 (4.00, 10.00)  4.00 (2.00, 16.00) |
| 240 mg fasted  Day 1  Day 3 | 8.81 (6.82–11.38)  9.87 (7.78–12.21) | 0.71 (0.52–0.96)  0.79 (0.62–1.02) | 0.19 (0.13–0.28)  0.22 (0.14–0.35) | 4.07 (4.00, 12.00)  5.03 (4.00, 20.00) |
| 300 mg standard meal  Day 1  Day 3 | 9.66 (6.42–14.54)  10.95 (7.47–16.05) | 0.89 (0.77–1.03)  1.08 (0.72–1.63) | 0.13 (0.06–0.27)  0.20 (0.10–0.42) | 4.00 (4.00, 10.00)  4.00 (4.00, 6.00) |

AUC_(0-24)_, area under the plasma concentration vs time curve from 0 to 24 h; CI, confidence interval; C_max_, maximum plasma concentration; C_24h_, observed concentration at 24 h post-dose, max, maximum; min, minimum; MT, matrix minitablet; MT-12 h, MT with 80% release at 12 h; PK, pharmacokinetic; T_max_, time to C_max_.

**Online Resource II.** Summary of statistical analyses of plasma GSK2982772 PK parameters assessing relative bioavailability of the MT formulation (Part B)

|  | AUC_(0-inf)_ Ratio (95% CI) | C_max_ Ratio (95% CI) |
| --- | --- | --- |
| 120 mg MT-12 h fasted, day 3 vs day 1 (*N*=13) | 1.07 (1.00–1.15) | 0.96 (0.83–1.10) |
| 240 mg MT-12 h fasted, day 3 vs day 1 (*N*=16) | 1.14 (1.05–1.23) | 1.12 (0.93–1.35) |
| 300 mg MT-12 h (standard meal), day 3 vs day 1 (*N*=16) | 1.11 (0.88–1.41) | 1.22 (0.93–1.59) |

AUC_(0-inf)_, area under the plasma concentration vs time curve from 0 to infinity; CI, confidence interval; C_max_, maximum plasma concentration; MT, matrix minitablet; MT-12 h, MT with 80% release at 12 h; PK, pharmacokinetic.

**Online Resource III.** Summary of statistical analyses of plasma GSK2982772 PK parameters assessing relative bioavailability of the MM formulation (Part C)

|  | AUC_(0-t)_ (h∙μg/mL) | | C_max_ (μg/mL) | |
| --- | --- | --- | --- | --- |
|  | Geometric Mean | Ratio (90% CI) | Geometric Mean | Ratio (90% CI) |
| 240 mg MM-12 h fasted  240 mg IR fasted | 9.68  14.62 | 0.66 (0.62–0.71) | 0.92  2.94 | 0.31 (0.28–0.35) |
| 240 mg MM-12 h delayed fed (high-fat)  240 mg MM-12 h fasted | 9.20  9.68 | 0.91 (0.82–1.01) | 1.06  0.92 | 1.14 (0.94–1.38) |
| 480 mg MM-12 h delayed fed (standard)  480 mg MM-12 h fasted | 19.15  20.01 | 0.95 (0.87–1.03) | 1.55  2.01 | 0.77 (0.64–0.91) |
| 480 mg MM-12 h fed (standard)  480 mg MM-12 h fasted | 22.71  20.01 | 1.14 (1.05–1.23) | 3.15  2.01 | 1.57 (1.32–1.86) |

AUC_0-t_, area under the plasma concentration vs time curve for the dosing interval; CI, confidence interval; C_max_, maximum plasma concentration; IR, immediate release; MM, matrix monolithic; MM-12 h, MM with 80% release at 12 h; PK, pharmacokinetic.
